# Safety of primaquine given to people with G6PD deficiency: systematic review of prospective studies

**DOI:** 10.1186/s12936-017-1989-3

**Published:** 2017-08-22

**Authors:** Olalekan A. Uthman, Patricia M. Graves, Rachel Saunders, Hellen Gelband, Marty Richardson, Paul Garner

**Affiliations:** 1 Centre for Evidence Synthesis in Global Health, Department of Clinical Sciences, Liverpool School of Tropical Medicine, Liverpool, UK; 2 Warwick Centre for Applied Health Research and Delivery (WCAHRD), Division of Health Sciences, Warwick Medical School, University of Warwick, Coventry, CV4 7AL UK; 3 College of Public Health, Medical and Veterinary Sciences, James Cook University, Cairns, Australia; 4 Cochrane Infectious Disease Group, Liverpool School of Tropical Medicine, Pembroke Place, Liverpool, UK

**Keywords:** Malaria, Primaquine, Glucose-6-phosphate dehydrogenase (G6PD)

## Abstract

**Background:**

Haemolysis risk with single dose or short course primaquine was evaluated in glucose-6-phosphate dehydrogenase (G6PD) deficient people.

**Methods:**

Major electronic databases (to August 2016) were searched for single or short course 8-aminoquinolines (8-AQ) in (1) randomized comparisons against placebo in G6PD deficient people; and (2) observational comparisons in G6PD deficient compared to replete people. Two authors independently assessed eligibility, risk-of-bias, and extracted data.

**Results:**

Five randomized controlled trials and four controlled observational cohorts were included. In G6PD deficient individuals, high-dose (0.75 mg/kg) PQ resulted in lower average haemoglobin levels at 7 days (mean difference [MD] −1.45 g/dl, 95% CI −2.17 to −0.74, 2 trials) and larger percentage fall from baseline to day 7 (MD −10.31%, 95% CI −17.69 to −2.92, 3 trials) compared to placebo. In G6PD deficient compared to replete people, average haemoglobin was lower at 7 days (MD −1.19 g/dl, 95% CI −1.94 to −0.44, 2 trials) and haemoglobin change from baseline to day 7 was greater (MD −9.10%, 95% CI −12.55 to −5.65, 5 trials). One small trial evaluated mid-range PQ dose (0.4–0.5 mg/kg) in G6PD deficient people, with no difference detected in average haemoglobin at day 7 compared to placebo. In one cohort comparing G6PD deficient and replete people there was a greater fall with G6PD deficiency (MD −4.99%, 95% CI −9.96 to −0.02). For low-dose PQ (0.1–0.25 mg/kg) in G6PD deficient people, haemoglobin change from baseline was similar to the placebo group (MD 1.72%, 95% CI −1.89 to 5.34, 2 trials). Comparing low dose PQ in G6PD deficient with replete people, the average haemoglobin was lower in the G6PD deficient group at 7 days (−0.57 g (95% CI −0.97 to −0.17, 1 trial)); although change from baseline was similar (MD −1.45%, 95% CI −5.69 to 2.78, 3 trials).

**Conclusions:**

Falls in average haemoglobin are less marked with the 0.1 to 0.25 mg/kg PQ than with the 0.75 mg/kg dose, and severe haemolytic events are not common. However, data were limited and the evidence GRADE was low or very low certainty.

**Electronic supplementary material:**

The online version of this article (doi:10.1186/s12936-017-1989-3) contains supplementary material, which is available to authorized users.

## Background

**Fig. 1:**
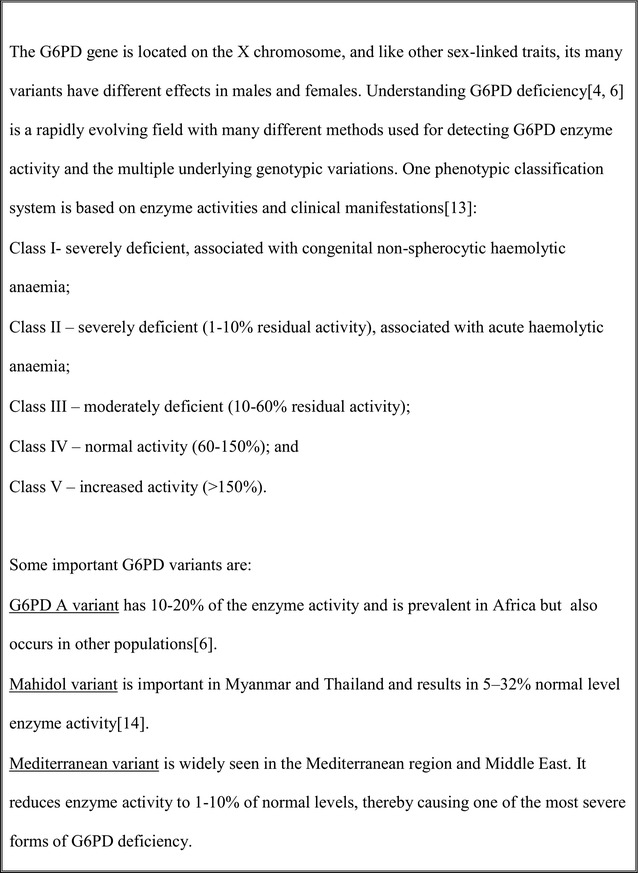
G6PD classification

Primaquine (PQ) has been used widely since the 1950s to prevent relapse from *Plasmodium vivax.* PQ also has a specific action on *Plasmodium falciparum* gametocytes, as does tafenoquine, another 8-aminoquinoline (8-AQ) [1]. This effect may reduce *P. falciparum* transmission to mosquitoes [2]. This reduction has no clinical benefit to the individual, but may prevent transmission of malaria and thus have public health benefits. However, 8-AQs can cause haemolysis in people with glucose-6-phosphate dehydrogenase (G6PD) deficiency. The detection and classification of the genotype and phenotype of G6PD deficiency is complicated and understanding is continually developing (Fig. 1; [3]). An estimated 400 million people worldwide carry G6PD gene mutations [4–6], and the relatively high prevalence of between 5 and 33% in the malaria endemic countries of sub-Saharan Africa and Asia [6] makes the drug potentially unsafe within these populations.

In 2010, the World Health Organization (WHO) re-affirmed a recommendation made many years previously for a single dose of 0.75 mg/kg of PQ to be administered with primary treatment for falciparum malaria to reduce transmission [7]. In reconsidering the risks, the WHO lowered the recommended single dose for gametocytocidal effect to 0.25 mg/kg in 2012, and limited PQ’s use to areas threatened by artemisinin resistance or areas of low endemicity approaching elimination [8]. Then in 2015, the policy recommendation was changed to recommend use in ‘low transmission areas’ and it was explicitly stated that G6PD testing was not required before single dose use [9].

The change in policy from 0.75 to 0.25 mg/kg was based partly on a WHO Evidence Review Group report [10, 11] summarizing published literature narratively, but did not use standard synthesis approaches to critically appraise the evidence. In light of the importance of the recommendation when implemented at scale, it could mean millions of people exposed to PQ or a related drug for the first time [12]. In this systematic review, a narrower objective was considered: to assess the risk and extent of haemolysis in people with G6PD deficiency given PQ or other 8-AQ, from existing research.

## Methods

### Inclusion criteria

*Study designs* Comparisons of single or short course 8-AQ versus placebo given to G6PD deficient people in randomized trials and controlled cohort designs were sought. This was supplemented with comparisons of 8-AQ given to G6PD deficient or G6PD replete people.

*Participants* Adults or children tested for G6PD deficiency.

*Intervention* Single dose or short course 8-AQ alone or added to malaria treatment. Since doses and schedules vary by country, any single dose regimens were included. The short course was defined as any regimen lasting up to 7 days. The results are expressed within two comparisons (PQ vs placebo in G6PD deficient people; and PQ in G6PD deficient people compared with G6PD replete people).

*Control* Comparison 1: placebo or no intervention. Comparison 2: PQ in G6PD replete individuals.

*Outcomes* Haemoglobin values at baseline and day 7. Absolute and percentage decline in haemoglobin.

*Information sources and search strategy* The following major electronic databases were searched using the criteria in Additional file 1 to August 2016: the Cochrane Infectious Diseases Group Specialized Register, the Cochrane Central Register of Controlled Trials (CENTRAL), MEDLINE, Scopus, Web of Science; EMBASE and LILACS. All relevant trials, regardless of language or publication status (published, unpublished, in press and in progress) were identified. The MIM Pan-African Malaria Conferences and the American Society of Tropical Medicine and Hygiene (ASTMH) (from 2004 to 2016) were also searched for relevant abstracts of unpublished trials, and researchers and other experts in the field of malaria chemotherapy were contacted. The reference lists of all retrieved articles were also reviewed [11].

*Study selection and data collection* RS and OU independently screened the results of the search strategy and retrieved the full text of all relevant citations. The inclusion criteria were independently applied using a spreadsheet and any discrepancies were discussed or referred to a third reviewer (PAG or PMG). RS and OU independently extracted data on study methods, participants, interventions and outcomes, and attempted to contact authors where data were missing.

*Risk of bias* RS and OU independently assessed the risk of bias for each study using predefined standards based on the Cochrane risk of bias tool for randomized controlled trials, and the EPOC criteria [15] for assessing risk of bias of non-randomized studies (Additional file 2). Each domain was categorized as ‘low’ risk of bias, ‘high’ risk of bias or ‘unclear’. Where the judgment was that the risk of bias was unclear, attempts were made to contact the authors for clarification. The quality of evidence was assessed using the GRADE approach [16].

*Summary measures* The results were stratified as high (0.75 mg/kg), medium (0.4–0.5 mg/kg) and low (0.1–0.3 mg/kg) dose of PQ.

Percentage change in haemoglobin (Hb) concentration from baseline was calculated as:

$$\begin{aligned}( {{\text{Hb post-treatment }}( {{\text{measured at day }}7}) - {\text{Hb pre-treatment }}( {\text{at baseline}})})/{\text{Hb pre-treatment }}( {\text{at baseline}}).\end{aligned}$$

This percentage change was categorized as: greater than −5%, greater than −10% and greater than −20%. Moderate and severe anaemia were defined as haemoglobin less than 8 g/dl and less than 5 g/dl, respectively [17–21]. Where the data were not provided, all recent study authors were contacted. The author of two trials [22, 23] provided additional individual patient data, which were included in the analysis.

The values were reported as:

- Mean difference in average haemoglobin values at day 7.
- Mean difference in percent haemoglobin value change between baseline and day 7.
- Relative risk of ≥5, ≥10 or ≥20% decline in haemoglobin concentration by day 7.
- Relative risk of moderate (≤8 g/dl) and severe (≤5 g/dl) anaemia at day 7.

The dichotomous outcomes were reported as risk ratios (RRs) and continuous outcomes as mean differences. Both measures are presented with 95% confidence intervals (CIs). When standard deviations were not reported [24], we imputed these values from comparatively sized trials [22].

*Synthesis of results* All eligible studies of primaquine were summarized and analysed using Review Manager 5.3. Data were presented in tables and meta-analyses performed when appropriate. In the absence of statistical heterogeneity, a fixed-effects model was used. A random-effects model was used where moderate heterogeneity was detected but it was deemed still reasonable to combine trials.

This systematic review was reported according to the Preferred Reporting Items for Systematic Reviews and Meta-analyses (PRISMA) guidelines [25, 26]. PRISMA checklist is provided in the Additional files 3. The review protocol was published a priori [3].

## Results

### Study selection and study characteristics

**Fig. 2:**
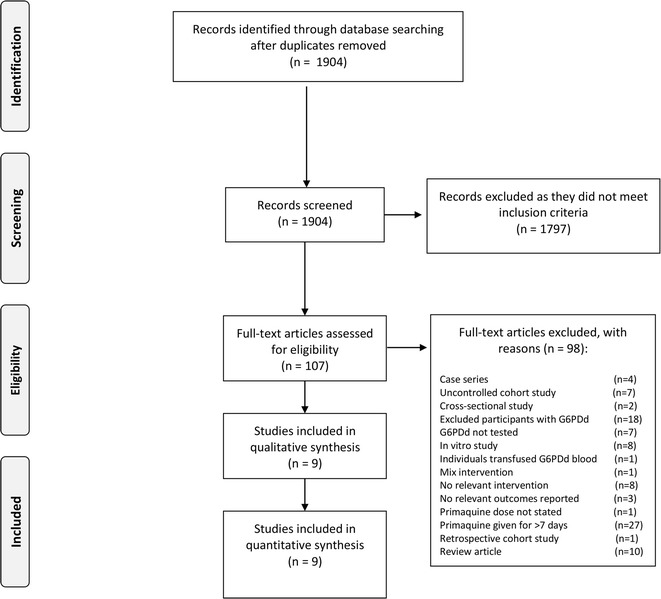
Study selection flow diagram

1904 unique citations were identified from the search (Fig. 2). 1797 were excluded after abstract screening and 98 after applying the inclusion criteria to the full text. We excluded 98 studies for the reasons given in the characteristics of excluded studies Additional File 4. Five randomized controlled trials [22–24, 27, 28] and four controlled observational cohorts [29–32], all published between 2004 and 2016, met the inclusion criteria (see Table 1). Eight studied primaquine, and one study available as a conference abstract studied tafenoquine. G6PD deficiency detection methods, phenotypes and genotypes are summarized in Table 2. Four studies reported that they used the fluorescent spot test (FST) for screening [27, 29–31], and one study used the rapid test rather than FST [28]. Studies also reported enzyme quantification and/or polymerase chain reaction to detect G202A/A376G mutation [22, 23, 27, 28] or Mahidol mutation [29, 30, 32]. The reported genotypes varied across studies, and G6PD activity was not always measured and not consistently reported, so no stratified analysis was possible.

**Table 1:** Summary of study participant characteristics

Study name	Study design	8-AQ duration	Dose	Adult dose (mg)^a^	Total adult dose (mg)	Malaria	Country	Setting	Population	Age	PQ compared to placebo in G6PD deficient people	PQ in G6PD deficient compared to G6PD replete people
Primaquine												
Krudsood [24]	Randomized control trial	7 days	30 mg	30	210	Yes	Thailand	Patients	Primary care urban	16–51 years	No	Yes
Shekalaghe [22]	Randomized control trial	Once	0.75 mg/kg	45	45	Yes	Tanzania	Residents	Rural	3–15 years	Yes	Yes
Shekalaghe [23]	Randomized control trial	Once	0.75 mg/kg	45	45	No	Tanzania	Residents	Rural	1–11 years	Yes	Yes
Eziefula [27, 33]	Randomized control trial	Once	0.1, 0.4 or 0.75 mg/kg	6, 24, 45	6, 24, 45	Yes	Uganda	Residents	Unknown	1–10 years	Yes	Yes
Kheng [30]	Open parallel clinical trial	Once	0.75 mg/kg	45	45	No	Cambodia	Healthcare centres		Adults and children	No	Yes
Bancone [29]	Cohort study	Once	0.25 mg/kg	15	15	No	North-Western Myanmar–Thailand border	Residents	Rural	Adults and children	No	Yes
Ley [31]	Cohort study	Once	0.75 mg/kg	45	45	Yes	Bangladesh	Healthcare	Rural	Adults and children	No	Yes
Mwaiswelo [28]	Randomized control trial	Once	0.25 mg/kg	15	15	Yes	Tanzania	Dispensary	Town	Men and non-pregnant, non-lactating women aged ≥1 year	Yes	Yes
Tafenoquine												
Rueangweerayut [32]	Cohort study	Once	100 mg (1.67 mg/kg), 200 mg (3.33 mg/kg), 300 mg (5.00 mg/kg)	100, 200, 300	100, 200, 300	No	Thailand	Laboratory	Research institute	18–45 years	No	Yes

^a^Adult dose—assuming 60 kg adult

**Table 2:** G6PD detection methods, phenotypes and genotypes

Study (country)	Methods	Results	
		Phenotype	Genotype
Krudsood [24] (Thailand)	Not reported	G6PD normal: 134 patients G6PD deficient: 7 patients	Not tested
Shekalaghe [22] (Tanzania)	PCR for G202A, A376G	G6PD normal: 77 patients G6PD deficient: 30 patients	G6PD wild type: 77 patients G6PD deficient heterozygous: 23 patients G6PD deficient hemizygous/homozygous: 7 patients
Shekalaghe [23] (Tanzania)	PCR for G202A, A376G	G6PD normal: 493 patients G6PD deficient: 69 patients	G6PD wild type: 493 patients G6PD deficient heterozygous: 47 patients G6PD deficient hemizygous/homozygous: 22 patients
Rueangweerayut [32] (Thailand)	PCR RFLP for Mahidol	G6PD normal (Mahidol negative with >80% G6PD activity): 6 patients G6PD deficient (G6PD enzyme activity 40–60% of normal): 6 patients	G6PD wild type: 6 patients G6PD deficient heterozygous: 6 patients
Eziefula [27, 33]^a^ (Uganda)	FST; PCR for G202A/A376G	G6PD normal: 373 patients G6PD deficient: 88 patients	G6PD wild type: 373 patients G6PD deficient heterozygous: 61 patients G6PD deficient hemizygous/homozygous: 27 patients
Kheng [30] (Cambodia)	FST; enzyme quantification; PCR for Mahidol, Mediterranean, Coimbra, Chinese-5, and Canton	G6PD normal: 57 patients G6PD deficient: 18 patients	G6PD wild type: 57 patients G6PD deficient hemizygous male: 14 patients G6PD deficient homozygous female: 1 patient G6PD deficient heterozygous female: 3 patients
Bancone [29] (Thai–Myanmar border)	FST; enzyme quantification; PCR RFLP for Mahidol. Chinese-4, Canton and Viangchan	G6PD normal: 1226 patients G6PD intermediate: 50 patients G6PD deficient: 124 patients	G6PD wild type: 1226 patients G6PD deficient hemizygous male: 39 patients G6PD deficient homozygous female: 5 patient G6PD deficient heterozygous female: 65 patients
Ley [31] (Bangladesh)	FST; enzyme quantification	G6PD normal: 169 Mild G6PD deficiency: 5 patients Severe G6PD deficiency: 1 patient	Not tested
Mwaiswelo [28] (Tanzania)	Rapid test; PCR for G202A, A376G	G6PD normal: 184 patients G6PD deficient: 33 patients	Males G6PD A wild type: 95 patients G6PD A− hemizygous: 15 patients
			Females G6PD AA and BA wild type: 83 patients G6PD A−A− homozygous: 5 patients G6PD AA− and BA−heterozygous: 22 patients

*FST* fluorescent spot test, *PCR* polymerase chain reaction, *RFLP* restriction fragment length polymorphism (RFLP)

^a^Only G6PD ‘normal’ participants based on the FST test; those not normal G6PD by FST were excluded and the genotyping was done on the remainder

*PQ compared to placebo in G6PD deficient individuals* Four randomized controlled trials reported haemoglobin levels [22, 23, 27, 28]. Three out of the four studies recruited only children and adolescents [22, 23, 27] and one recruited participants of all ages [28]. Three studies were conducted in individuals with confirmed malaria (*P. falciparum* [22, 27, 28]).

One study used high (0.75 mg/kg), medium (0.4–0.5 mg/kg) and low (0.1–0.25 mg/kg) dose PQ [27], two studies used only high dose PQ [22, 23], and one used low dose PQ [28].

*PQ in G6PD deficient compared to replete individuals* Five randomized controlled trials [22–24, 27, 28] and three controlled observational cohorts [29–31] reported haemoglobin levels after PQ treatment. One of the eight studies recruited only adults [24], and four recruited participants of all ages [24, 28–31]. Five studies were conducted in people with confirmed malaria: three of *P. falciparum* [22, 27, 28] and two of *P. vivax* [24, 31]. One of the eight studies [24] administered PQ as a 7-day course and the remaining seven studies used single dose PQ [22, 23, 27–31]. Five studies used high dose PQ [22, 23, 27, 30, 31], two used medium dose PQ [24, 27] and three used low dose PQ [27–29].

One controlled observational cohort evaluated 100, 200 and 300 mg single dose tafenoquine [32]. This study recruited adults without malaria.

**Fig. 3:**
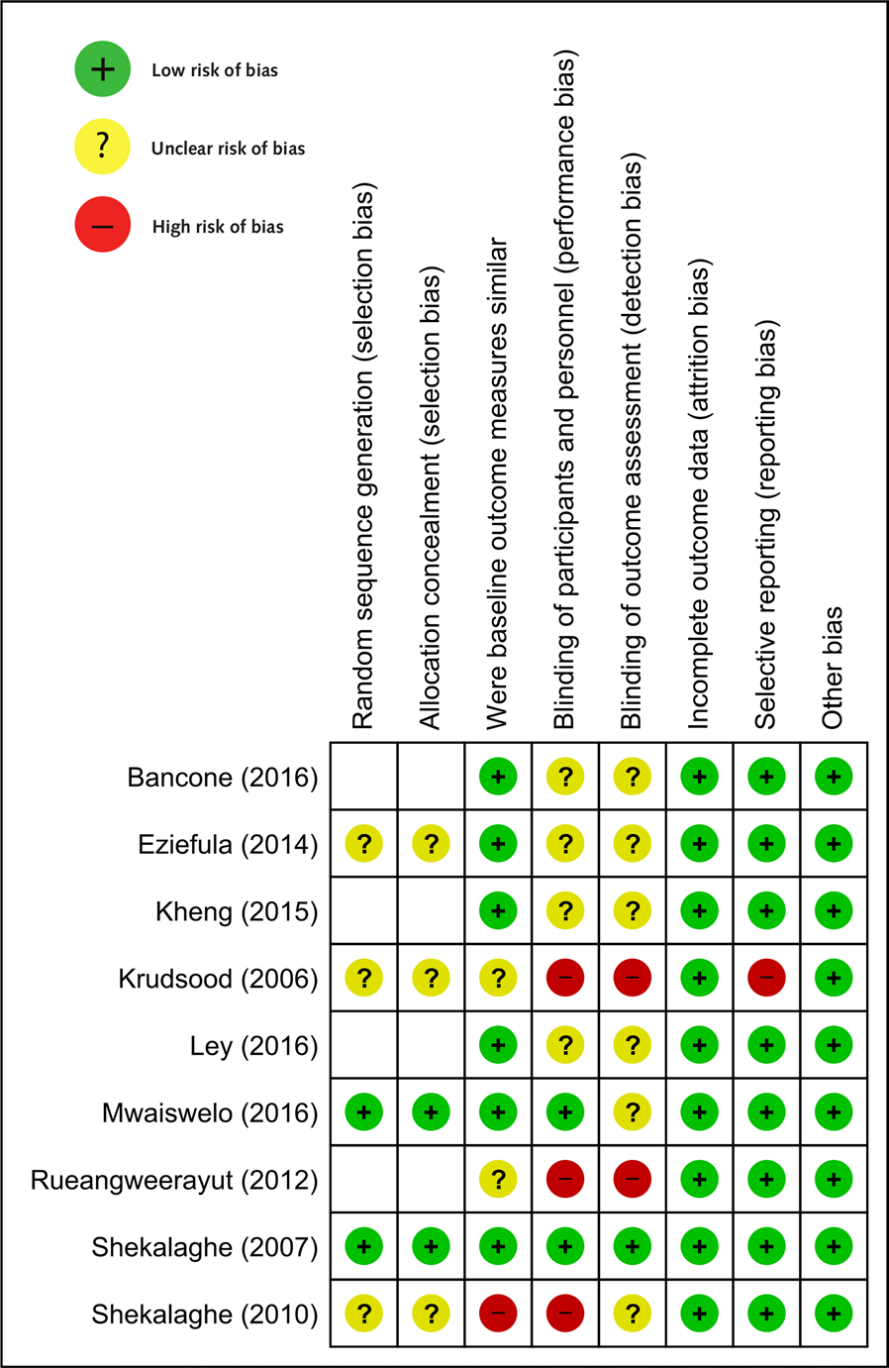
Risk of bias of included studies

*Risk of bias assessment* The risk of bias of the nine included studies is summarized in Fig. 3.

*Baseline characteristics* Six studies were classed as low risk of bias [22, 27–31]; in two it was unclear; and in one there were baseline differences that were likely to influence the results [22].

*Blinding of outcome assessors* One study was low risk (an RCT) [22]; six studies were classed as unknown risk of bias; two studies did not blind outcome assessors [24, 32].

*Attrition and incomplete outcome data* All studies were deemed to be at low risk of attrition bias.

*Reporting bias and selective reporting* One study was at high risk of reporting bias as stated outcomes were not reported within the results [24]. Of the five trials described as randomized [22–24, 27, 28], risk of bias due to random sequence generation and allocation concealment was low in two studies [22, 28] and unclear in the remaining three studies [23, 24, 27].

### Certainty of evidence

Our assessment of the quality of evidence using the GRADE approach is presented in summary of findings tables (Additional files 5, 6, 7, 8, 9, 10).

#### High dose (0.75 mg/kg) PQ

*PQ compared to placebo in G6PD deficient people* Three randomized trials [22, 23, 27] report 0.75 mg/kg PQ versus placebo in people with G6PD deficiency, all part of larger trials. Numbers with G6PD deficiency were small, and one trial excluded those with severe deficiency [27]. Two trials were considered low risk of bias [22, 27], while one was at high risk of bias due to large baseline differences in haemoglobin concentration between groups [23].

*Average haemoglobin values at day 7* Two trials reported this [22, 23], and found haemoglobin to be lower with PQ (MD −1.45 g/dl, 95% CI −2.17 to −0.74, 93 participants, *low certainty evidence*, Fig. 4.1).

**Fig. 4:**
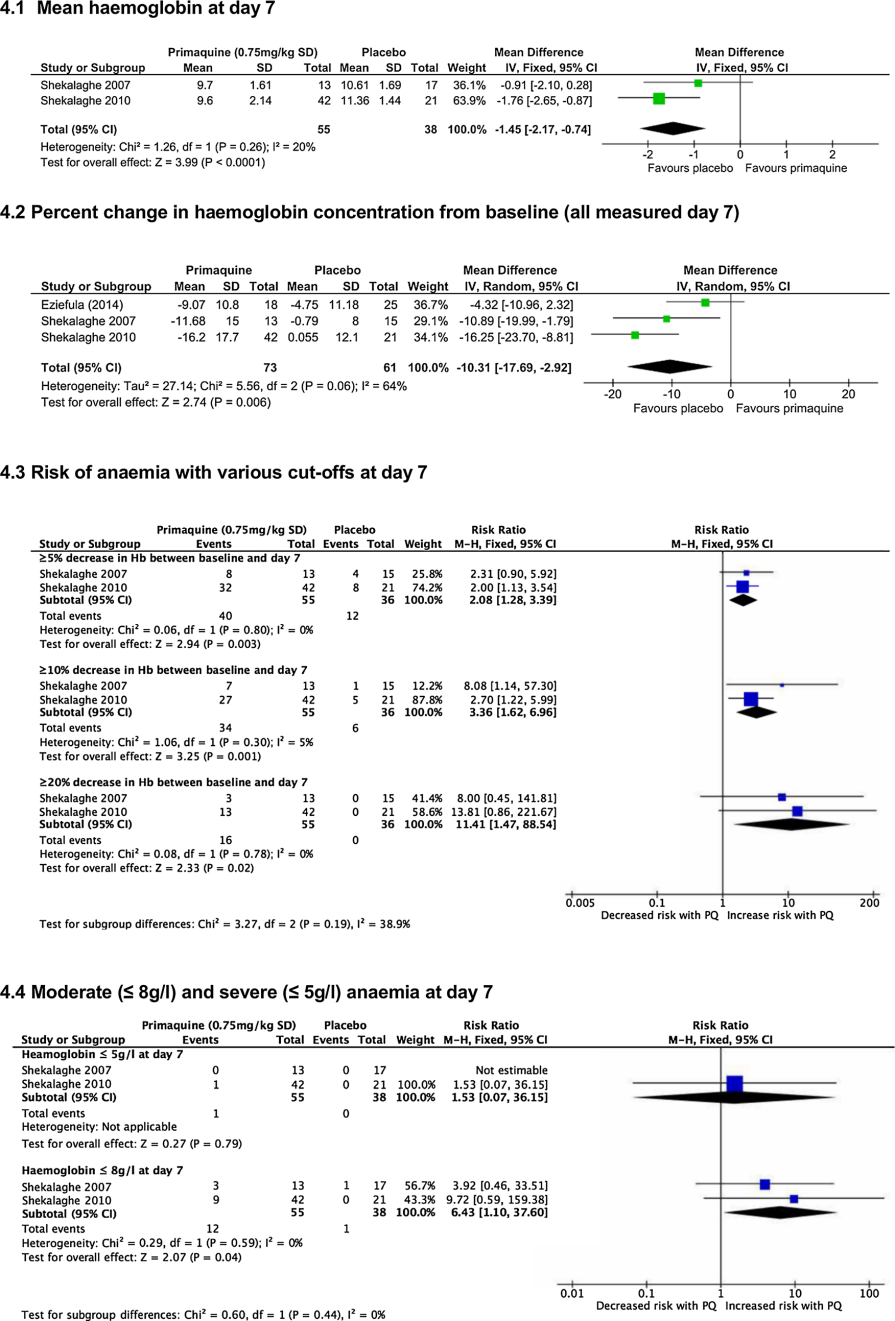
High dose PQ (0.75 mg/kg) compared to placebo in G6PD deficient people

*Percent haemoglobin value change between baseline and day* Three trials reported this [22, 23, 27], and haemoglobin fell further in individuals treated with PQ (mean difference −10.31%, 95% CI −17.69 to −2.92, *I* ^*2*^ = 64%, 134 participants, *low certainty evidence,* Fig. 4.2).

*Risk of anaemia* Two trials reported this [22, 23], with an 11-fold increase in the proportion of individuals with ≥20% fall (*low certainty evidence*, Fig. 4.3).

*Moderate and severe anaemia at day 7* Two trials reported this [22, 23], with an increased risk of anaemia defined by ≤8 g/dl by day 7, with one individual with Hb ≤5 g/dl (*low certainty evidence*, Fig. 4.4).

*PQ in G6PD deficient compared to G6PD replete people* Five controlled observational cohorts evaluated changes in haemoglobin when 0.75 mg/kg PQ was given to people with and without G6PD deficiency [22, 23, 27, 30, 31].

*Average haemoglobin values at day 7* Two trials reported this [22, 23] and on average, haemoglobin was lower with G6PD deficiency (MD −1.19 g/dl, 95% CI −1.94 to −0.44, 493 participants, *very low certainty evidence*, Fig. 5.1).

**Fig. 5:**
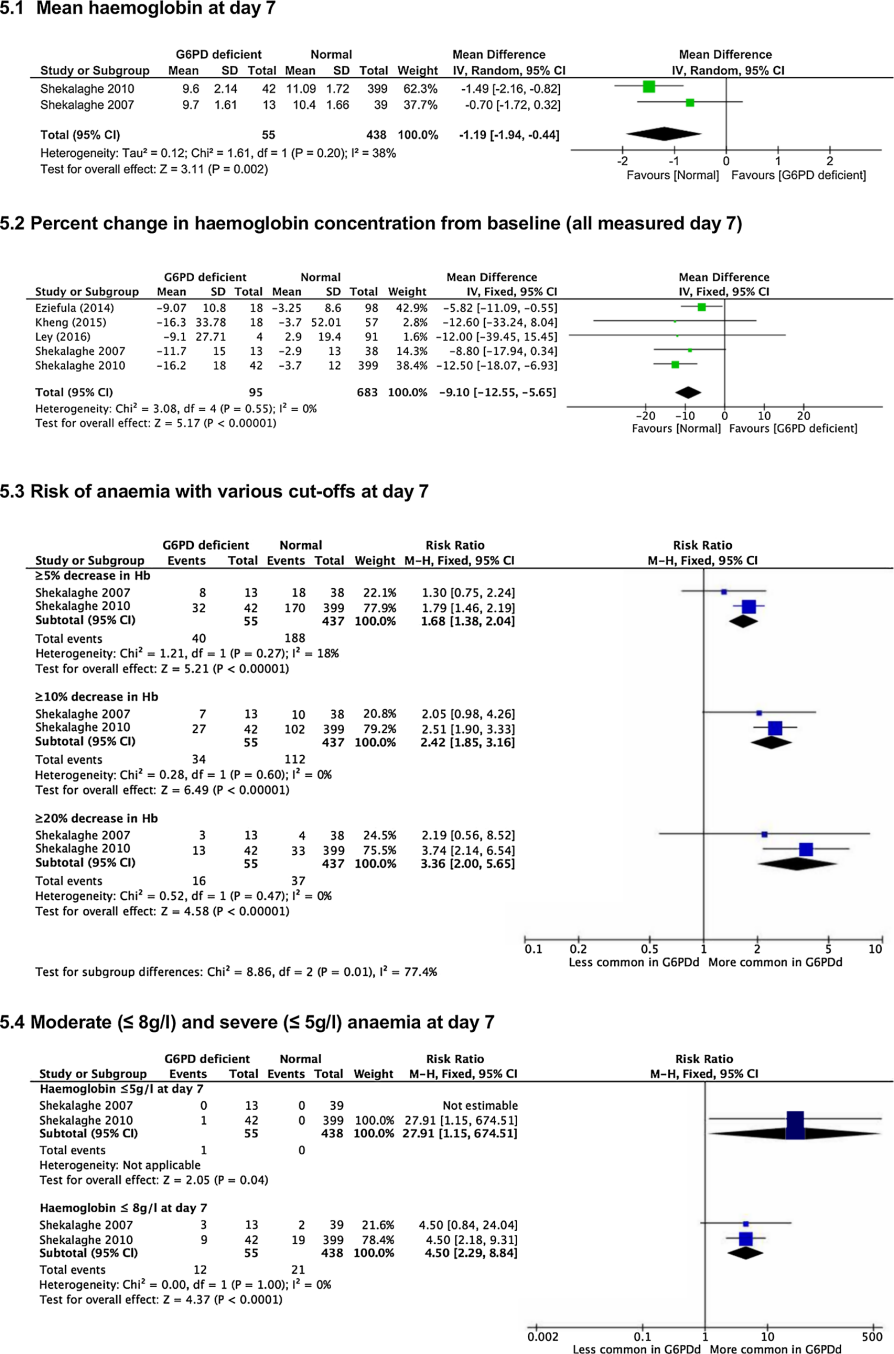
High dose PQ (0.75 mg/kg) in G6PD deficient compared to G6PD replete people

*Percent haemoglobin value change between baseline and day 7* Five trials reported this [22, 23, 27, 30, 31], and haemoglobin fell further in G6PD deficient people (MD −9.10%, 95% CI −12.55 to −5.65, *I* ^*2*^ = 0%, 778 participants, *very low certainty evidence,* Fig. 5.2).

*Risk of anaemia* Two trials reported this [22, 23] and there was a 3.4-fold increase in risk for ≥20% fall (*very low certainty evidence,* Fig. 5.3).

*Moderate and severe anaemia at day 7* Two trials reported this [22, 23] and there was increased risk of anaemia defined as ≤ 8 g/dl by day 7, with one individual with Hb ≤5 g/dl (Fig. 5.4).

#### Mid-range dose (0.4–0.5 mg/kg)

*PQ compared to placebo in G6PD deficient people* One trial [27] evaluated 0.4 mg/kg PQ. This trial did not report haemoglobin at day 7 or risk of anaemia.

**Fig. 6:**
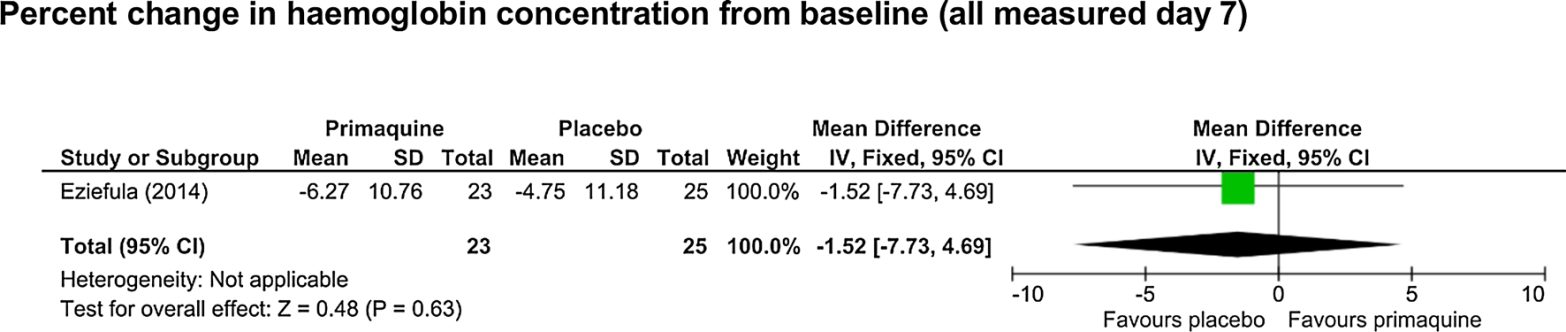
Mid-range dose (0.4–0.5 mg/kg) PQ compared to placebo in G6PD deficient people

*Percentage haemoglobin value change between baseline and day 7* One trial [27] reported this, and no difference was seen (MD −1.52%, 95% CI −7.73 to 4.69, one study, 48 participants, *low certainty evidence,* Fig. 6).

*PQ in G6PD deficient compared to G6PD replete people* Two controlled cohort studies evaluated changes in haemoglobin with a mid-range dose of PQ given to G6PD deficient and G6PD replete people [24, 27]. The doses used in these studies ranged from 0.4 to 0.5 mg/kg. The two trials did not report risk of anaemia.

*Average haemoglobin values at day 7* One trial [24] reported this, which was lower in people with G6PD deficiency (MD −3.92 g, 95% CI −4.93 to −2.91, 71 participants, *very low certainty evidence*, Fig. 7.1).

**Fig. 7:**
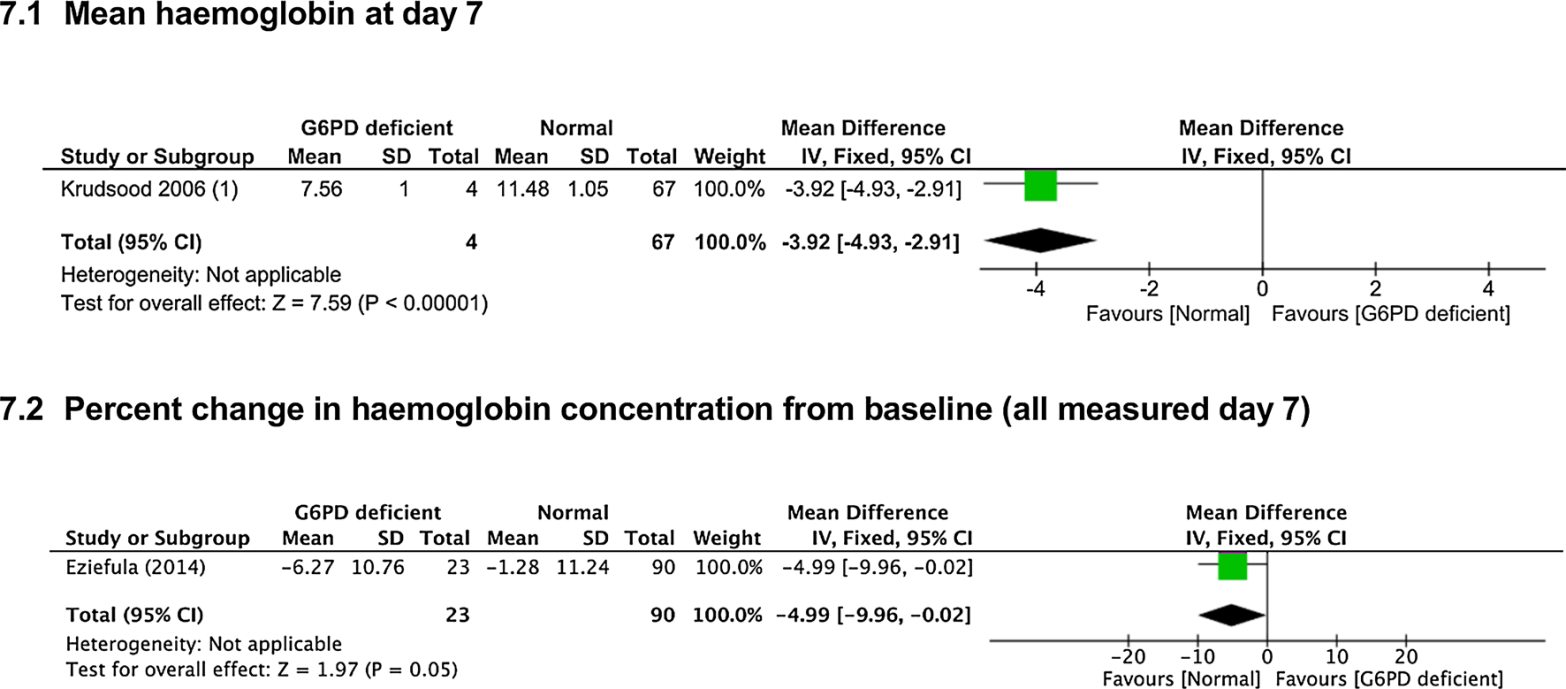
Mid-range dose (0.4–0.5 mg/kg) PQ in G6PD deficient compared to G6PD replete people

*Percent haemoglobin value change between baseline and day 7* One trial [27] reported this and found greater % change in G6PD deficient people (MD −4.99%, 95% CI −9.96 to −0.02, 113 participants, *very low certainty evidence*, Fig. 7.2).

#### Low dose (0.1–0.25 mg/kg)

*PQ compared to placebo in G6PD deficient people* Two trials [27, 28] provide data for this, using a single dose of PQ versus placebo in people with G6PD deficiency. The doses in these studies ranged from 0.1 to 0.25 mg/kg. These trials did not report mean haemoglobin at follow-up or risk of anaemia.

**Fig. 8:**
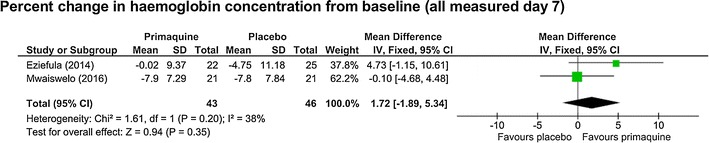
Low dose (0.1–0.25 mg/kg) PQ compared to placebo in G6PD deficient people

*Percent haemoglobin value change between baseline and day 7* Two trials reported this [23, 26], with no difference seen (MD 1.72%, 95% CI −1.89 to 5.34, *I* ^*2*^ = 38%, two studies, 89 participants, *low certainty evidence,* Fig. 8).

*PQ in G6PD deficient compared to G6PD replete people* Three controlled observational comparisons report doses ranging from 0.1 to 0.25 mg/kg [27–29]. The three trials did not report risk of anaemia.

*Average haemoglobin values at day 7* One trial [29] reported this, which was lower in G6PD deficient people (MD −0.57 g/dl, 95% CI −0.97 to −0.17, 830 participants, *very low certainty evidence*, Fig. 9.1).

**Fig. 9:**
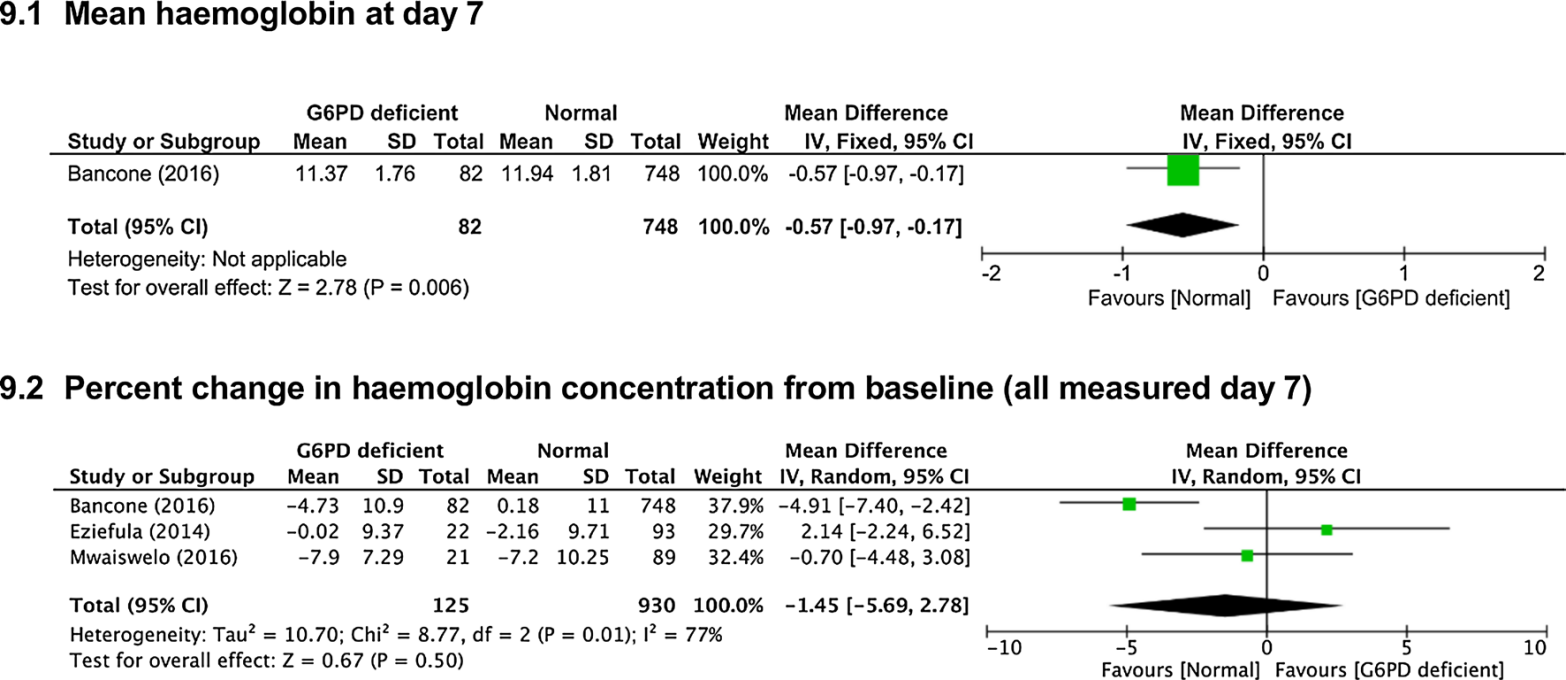
Low dose (0.1–0.25 mg/kg) PQ in G6PD deficient compared to G6PD replete people

*Percent haemoglobin value change between baseline and day 7* Three controlled observational comparisons reported this [22, 23, 26] and no difference was seen (MD −1.45% g/dl, 95% CI −5.69 to 2.78, three studies, 1055 participants, *very low certainty evidence,* Fig. 9.2).

#### Tafenoquine (Table 3)

*Tafenoquine in G6PD deficient compared to G6PD replete people* One controlled observational cohort compared average maximum fall in haemoglobin values in G6PD deficient and G6PD replete people given tafenoquine. 300 mg (5 mg/kg) of tafenoquine produced a greater average maximum decline in haemoglobin over an average of 9 days in G6PD deficient than in G6PD replete individuals (Table 3). The average maximal decline in haemoglobin following 100 mg (1.67 mg/kg) and 200 mg (3.33 mg/kg) of TQ did not demonstrate a difference.

**Table 3:** Changes in haemoglobin with different doses of tafenoquine (TQ) [32]

	TQ 100 mg		TQ 200 mg		TQ 300 mg	
	G6PD normal	G6PD deficiency	G6PD normal	G6PD deficiency	G6PD normal	G6PD deficiency
Number of participants	6	6	6	6	6	6
Average maximum decline in haemoglobin (g/dl) from baseline	−1.8	−1.28	−1.23	−1.83	−0.75	−2.78
Average day of maximum decline	10	10	10	10	10	9

## Discussion

In this review, comparative information about effects of PQ (and tafenoquine) on haemoglobin in G6PD deficient people was summarized. The aim was to quantify the risk of adverse effects in those given single dose primaquine to reduce infectiousness of *P. falciparum.* Few trials to date have addressed this issue directly, so indirect evidence from trials that had the main aim of assessing impact of 8-AQ on *P. falciparum* gametocytes was sought. Whilst these trials generate some useful information on haemoglobin, they were not necessarily powered for the outcomes studied here.

The nine trials in this review were conducted with varying levels of methodological rigor. Nevertheless, with the 0.75 mg/kg single dose in G6PD deficient people there is an average 1.45 g/dl greater fall in haemoglobin by day 7 compared to placebo, and risks of anaemia (20% drop in haemoglobin) are increased 11-fold. For the mid-range dose of PQ in G6PD deficient compared to G6PD replete people, one trial reported a greater fall in haemoglobin of 3.92 g/dl by day 7.

For the currently recommended low dose of 0.25 mg/kg, there was no difference in the percentage fall in haemoglobin by day 7 in G6PD deficient people given low dose PQ compared to placebo. However, in one trial of PQ given to G6PD deficient compared with G6PD replete people, the average haemoglobin value at day 7 was 0.57 g/dl lower in the deficient group. No difference was seen in percent change in haemoglobin between G6PD deficient and replete people. These data are somewhat reassuring, but the total number of the trials and people in the low dose (0.1 to 0.25 mg/kg) is few, and large falls in a few individuals cannot be excluded.

The lack of sufficient data prevented any analysis stratified by G6PD type and enzyme activities. The reported genotypes varied across studies. It should be noted that one trial contributing to both comparisons [27, 33] included only G6PD ‘normal’ participants based on the FST test, but further genotyping showed wide variation in G6PD genotypes. Therefore, this study likely underestimated the effect in G6PD deficient participants. Individual patient data meta-analysis is needed to explore variation due to phenotypic G6PD activity, genotype or applied diagnostic method, which is beyond the scope of the present analysis.

The enduring uncertainty surrounding safety, shown clearly by low or very low certainty of the evidence, should prompt further research on the effect of lower doses of PQ in people with G6PD deficiency, including examining effects on the most prevalent genotypes. Only this additional information will enable policies to be based on an adequate evidence base and reassure national and local programme managers that the drug is safe to be used without prior G6PD testing.

Several studies on the safety of the currently recommended single low dose of 0.25 mg/kg PQ in combination with artemisinin combination therapy in G6PD deficient individuals are ongoing (clinicaltrials.gov NCT02174900; NCT02654730; NCT02535767). These studies will help refine our understanding of the safety of PQ in G6PD deficient individuals. Enhanced and continuing pharmacovigilance is needed to fully characterize the safety of PQ in the range of G6PD deficiency variants [34, 35].

## Conclusions

The evidence assessed for this systematic review suggests that the lower single dose (0.1 to 0.25 mg/kg) of PQ given with the goal of reducing transmission of *P. falciparum* is less likely to cause haemolytic effects in people with G6PD deficiency than the previous 0.75 mg/kg dose, and that severe haemolytic events are not very common. However, the evidence is based on few trials and a small number of people. The roll-out and scale-up of the 0.25 mg/kg dose in countries without prior experience would greatly benefit from more reliable data demonstrating the safety of this dose.

## Additional files

**Additional file 1.** Search strategy.

**Additional file 2.** Criteria for risk of bias assessment.

**Additional file 3.** PRISMA checklist.

**Additional file 4.** Characteristics of excluded studies.

**Additional file 5.** GRADE summary of findings table, high dose PQ (0.75 mg/kg) compared to placebo in G6PD deficient people.

**Additional file 6.** GRADE summary of findings table, high dose PQ (0.75 mg/kg) in G6PD deficient compared to G6PD replete people.

**Additional file 7.** GRADE summary of findings table, mid-range dose (0.4–0.5 mg/kg) PQ compared to placebo in G6PD deficient people.

**Additional file 8.** GRADE summary of findings table, mid-range dose (0.4–0.5 mg/kg) PQ in G6PD deficient compared to G6PD replete people.

**Additional file 9.** GRADE summary of findings table, low dose (0.1–0.25 mg/kg) PQ compared to placebo in G6PD deficient people.

**Additional file 10.** GRADE summary of findings table, low dose (0.1–0.25 mg/kg) PQ in G6PD deficient compared to G6PD replete people.
