# Supplementary material for: Safety of primaquine given to people with G6PD deficiency: systematic review of prospective studies
Source: Malar J. 2017 Aug 22;16:346. doi: 10.1186/s12936-017-1989-3 (PMC5568268; doi:10.1186/s12936-017-1989-3)
Supplement: Supplementary file 1 — Additional file 1. Search strategy. [file 12936_2017_1989_MOESM1_ESM.docx]

## Additional file 1. Search strategy

| Box 1. MEDLINE search strategy  1 antimalarials  2 8-aminoquinolone  3 primaquine  4 1 OR 2 OR 3  5 g-6-pd  6 glucose 6 phosphate dehydrogenase  7 g6pd deficiency  8 glucosephosphate dehydrogenase deficiency  9 5 OR 6 OR 7 OR 8  10 4 AND 9  11 haemoly*  12 hemoly*  13 11 OR 12  14 primaquine-sensitiv*  15 13 OR 14  16 Primaquine*  17 sensitive*  18 16 AND 17  19 15 OR 18  20 19 AND 4 |
| --- |
