# Supplementary material for: Safety of primaquine given to people with G6PD deficiency: systematic review of prospective studies
Source: Malar J. 2017 Aug 22;16:346. doi: 10.1186/s12936-017-1989-3 (PMC5568268; doi:10.1186/s12936-017-1989-3)
Supplement: Supplementary file 2 — Additional file 2. Criteria for risk of bias assessment. [file 12936_2017_1989_MOESM2_ESM.docx]

## Additional file 2. Criteria for risk of bias assessment

| - Was the allocation sequence adequately generated? - Was the allocation adequately concealed? - Were baseline outcome measurements similar? - Were incomplete outcome data adequately addressed? - Was knowledge of the allocated interventions adequately prevented during the study? - Was the study free from selective outcome reporting? - Was the study free from other risks of bias? |
| --- |
