# Supplementary material for: Safety of primaquine given to people with G6PD deficiency: systematic review of prospective studies
Source: Malar J. 2017 Aug 22;16:346. doi: 10.1186/s12936-017-1989-3 (PMC5568268; doi:10.1186/s12936-017-1989-3)
Supplement: Supplementary file 4 — Additional file 4. Characteristics of excluded studies. [file 12936_2017_1989_MOESM4_ESM.docx]

## Additional file 4. Characteristics of excluded studies

| **Study** | **Reason for exclusion** |
| --- | --- |
| Beutler, E.(1954a) | G6PD deficiency not tested |
| Beutler, E.(1954b) | G6PD deficiency not tested |
| Dern, R. J.(1954a) | Primaquine given for more than 7 days |
| Dern, R. J.(1954b) | Primaquine given for more than 7 days |
| Beutler, E.(1955a) | G6PD deficiency not tested |
| Beutler, E.(1955b) | G6PD deficiency not tested |
| Dern, R. J.(1955) | Primaquine given for more than 7 days |
| Alving, A. S.(1956) | In vitro study |
| Alving, A. S.(1958) | No relevant intervention |
| Flanagan, C. L.(1958) | Primaquine given for more than 7 days |
| Schrier, S. L.(1958) | G6PD deficiency not tested |
| Schrier, S. L.(1959) | In vitro study |
| Alving, A. S. (1960) | Primaquine given for more than 7 days |
| Charles, L. J. (1960) | No usable outcomes reported |
| Gilles, H. M.(1961) | Cross-sectional study |
| Kellermeyer, R. W.(1961a) | In vitro study |
| Kellermeyer, R. W.(1961b) | In vitro study |
| Tarlov, A. R.(1961) | Primaquine given for more than 7 days |
| Gervais, P.(1962) | In vitro study |
| Allison, A. C.(1963) | Review article |
| Prins, H. K.(1963) | Primaquine given for more than 7 days |
| Brewer, G. J.(1964a) | In vitro study |
| Brewer, G. J.(1964b) | In vitro study |
| Berry, D. H.(1965) | Case series |
| Ezra, R.(1965) | In vitro study |
| Newbern, B. L. (1966) | No relevant intervention |
| Brewer, G. J.(1967) | Primaquine given for more than 7 days |
| Salvidio, E.(1967) | Individuals transfused G6PDd blood |
| Abeyaratne, K. P.(1968) | Retrospective cohort study |
| Pannaccuilli, I.(1969) | Primaquine given for more than 7 days |
| Aung. T B.(1970) | Primaquine given for more than 7 days |
| McCurdy. P. R.(1971) | Review article |
| Ebisawa, I.(1972) | Primaquine given for more than 7 days |
| Elktara, A. M. (1972) | Review article |
| Motulsky, A. G.(1972) | Review article |
| Willerson, D., Jr.(1972) | Primaquine given for more than 7 days |
| Charoenlarp, P.(1973) | Primaquine given for more than 7 days |
| DiPalma, J. R.(1973) | Review article |
| McCurdy. P. R.(1973) | No relevant intervention |
| Bronshtein, A. M.(1975) | Primaquine given for more than 7 days |
| Shcherbakov, A. M.(1975) | Primaquine given for more than 7 days |
| Ozeretskovskaia, N. N.(1976) | Primaquine given for more than 7 days |
| Everett, W. D.(1977) | Primaquine given for more than 7 days |
| Saenko, G. P.(1977) | No relevant intervention |
| Clyde, D. F.(1981) | Review article |
| Reilly, Q. (1982) | G6PD deficiency not tested |
| Vaidya, A. B.(1983) | Review article |
| Goldsmid, J. M.(1984) | Review article |
| Grimmond, T. R.(1984) | G6PD deficiency not tested |
| Cheng, Z. F. (1986) | No usable outcomes reported |
| Wong, H. B.(1986) | Review article |
| Hadad Melendez, P.(1991) | No relevant intervention |
| Reeve, P. A.(1992) | Case series |
| Bangchang, K. N.(1994) | Primaquine given for more than 7 days |
| Myat Phone, Kyaw(1994) | Mix intervention, cannot extract outcomes on single dose |
| Bouma, M. J.(1995) | Primaquine dose not stated |
| Menendez Capote, R.(1997) | Primaquine given for more than 7 days |
| Gogtay, N. J. (1998) | Excluded participants with G6PD deficiency |
| Gogtay, N. J.(1999) | No relevant outcomes reported |
| Rowland, M.(1999) | Excluded participants with G6PD deficiency |
| Buchachart, K.(2001) | Primaquine given for more than 7 days |
| Baird, J. K.(2003) | Primaquine given for more than 7 days |
| Silachamroon, U(2003) | Primaquine given for more than 7 days |
| Carr, M. E., Jr.(2005) | Case series |
| Santana, M. S.(2007) | Primaquine given for more than 7 days |
| Leslie, T.(2008) | Primaquine given for more than 7 days |
| Carmona-Fonseca, J.(2009) | Excluded participants with G6PD deficiency |
| Kondrashin, A. V.(2010) | Review article |
| Ramos Junior, W.M.(2010) | Case series |
| Carter, N.(2011) | No relevant intervention |
| Ferreira, M. E. S.(2011) | No relevant intervention |
| Betuela, I(2012) | Excluded participants with G6PD deficiency |
| Eziefula, A. C.(2013) | Excluded participants with G6PD deficiency |
| Khim, N.(2013) | No relevant intervention |
| Llanos-Cuentas, A.(2013) | Primaquine given for more than 7 days |
| Pasaribu, A. P.(2013) | Primaquine given for more than 7 days |
| Santana, M. S.(2013) | Cross-sectional study |
| Kondrashin, A.(2014) | Primaquine given for more than 7 days |
| Diawara (2014) | Excluded participants with G6PD deficiency |
| Dicko (2016) | Excluded participants with G6PD deficiency |
| Eziefula 2014 (lancet) | Excluded participants with G6PD deficiency |
| Eziefula (2014) | Excluded participants with G6PD deficiency |
| Goncalves (2016) | Excluded participants with G6PD deficiency |
| Green (2014) | Excluded participants with G6PD deficiency |
| Jittamala (2015) | Excluded participants with G6PD deficiency |
| John (2016) | Excluded participants with G6PD deficiency |
| Kheng (2015) | Excluded participants with G6PD deficiency |
| Ley (2016) | Excluded participants with G6PD deficiency |
| Llanos-Cuentas (2014) | Excluded participants with G6PD deficiency |
| Moore (2014) | Excluded participants with G6PD deficiency |
| Mwaiswelo (2016) | Excluded participants with G6PD deficiency |
| Nelman (2015) | Excluded participants with G6PD deficiency |
| Pukrittayakamee (2014) | Excluded participants with G6PD deficiency |
| Spring (2015) | Excluded participants with G6PD deficiency |
