# Supplementary material for: Safety of primaquine given to people with G6PD deficiency: systematic review of prospective studies
Source: Malar J. 2017 Aug 22;16:346. doi: 10.1186/s12936-017-1989-3 (PMC5568268; doi:10.1186/s12936-017-1989-3)
Supplement: Supplementary file 5 — Additional file 5. GRADE summary of findings table, high dose PQ (0.75 mg/kg) compared to placebo in G6PD deficient people. [file 12936_2017_1989_MOESM5_ESM.docx]

## Additional file 5: GRADE Summary of findings table, high dose PQ (0.75 mg/kg) compared to placebo in G6PD deficient people

| Outcomes | **Anticipated absolute effects^*^** (95% CI) | | Relative effect (95% CI) | № of participants  (studies) | Quality of the evidence (GRADE) |
| --- | --- | --- | --- | --- | --- |
|  | **Risk with Individuals with G6PD deficiency given placebo** | **Risk with Individuals with G6PD deficiency given Primaquine** |  |  |  |
| Mean values of haemoglobin at day 7 | Mean Hb was **10.99** | MD was 1.45 lower (2.17 lower to 0.74 lower) | - | 93 (2 RCTs) | ⨁⨁◯◯ LOW ^a^ |
| Percentage change in haemoglobin concentration from baseline (measured at day 7 | Mean change in Hb was **-1.83** | MD was 10.31 lower (17.69 lower to 2.92 lower) | - | 134 (3 RCTs) | ⨁⨁◯◯ LOW ^b,c^ |
| ≥5% decline in haemoglobin at day 7 | 333 per 1,000 | **693 per 1,000** (427 to 1,000) | **RR 2.08** (1.28 to 3.39) | 91 (2 RCTs) | ⨁⨁◯◯ LOW ^a^ |
| ≥10% decline in haemoglobin at day 7 | 167 per 1,000 | **560 per 1,000** (270 to 1,000) | **RR 3.36** (1.62 to 6.96) | 91 (2 RCTs) | ⨁⨁◯◯ LOW ^a^ |
| ≥20% decline in haemoglobin at day 7 | 0 per 1,000 | **0 per 1,000** (0 to 0) | **RR 11.41** (1.47 to 88.54) | 91 (2 RCTs) | ⨁⨁◯◯ LOW ^a^ |
| Rate of moderate Individuals with moderate (≤ 5g/l) anaemia at day 7 | 0 per 1,000 | **0 per 1,000** (0 to 0) | **RR 1.53** (0.07 to 36.15) | 93 (2 RCTs) | ⨁⨁◯◯ LOW ^a^ |
| Rate of moderate Individuals with severe (≤ 8 g/l) anaemia at day 7 | 26 per 1,000 | **169 per 1,000** (29 to 989) | **RR 6.43** (1.10 to 37.60) | 93 (2 RCTs) | ⨁⨁◯◯ LOW ^a^ |

| ***The risk in the intervention group** (and its 95% confidence interval) is based on the assumed risk in the comparison group and the **relative effect** of the intervention (and its 95% CI).   **CI:** Confidence interval; **MD:** Mean difference; **RR:** Risk ratio |
| --- |
| **GRADE Working Group grades of evidence** **High quality:** We are very confident that the true effect lies close to that of the estimate of the effect **Moderate quality:** We are moderately confident in the effect estimate: The true effect is likely to be close to the estimate of the effect, but there is a possibility that it is substantially different **Low quality:** Our confidence in the effect estimate is limited: The true effect may be substantially different from the estimate of the effect **Very low quality:** We have very little confidence in the effect estimate: The true effect is likely to be substantially different from the estimate of effect |

a. Imprecision rated very serious as small number of studies, smaller than the optimal information size

b. Inconsistency rated serious as there was considerable heterogeneity in treatment effect estimates (I² > 50%)

c. Imprecision rated serious as small number of studies, smaller than the optimal information size
