# Supplementary material for: Safety of primaquine given to people with G6PD deficiency: systematic review of prospective studies
Source: Malar J. 2017 Aug 22;16:346. doi: 10.1186/s12936-017-1989-3 (PMC5568268; doi:10.1186/s12936-017-1989-3)
Supplement: Supplementary file 6 — Additional file 6. GRADE summary of findings table, high dose PQ (0.75 mg/kg) in G6PD deficient compared to G6PD replete people. [file 12936_2017_1989_MOESM6_ESM.docx]

## Additional file 6: GRADE Summary of findings table, high dose PQ (0.75 mg/kg) in G6PD deficient compared to G6PD replete people

| Outcomes | **Anticipated absolute effects^*^** (95% CI) | | Relative effect (95% CI) | № of participants  (studies) | Quality of the evidence (GRADE) |
| --- | --- | --- | --- | --- | --- |
|  | **Risk with Individuals given primaquine without G6PD deficiency** | **Risk with Individuals given primaquine with G6PD deficiency** |  |  |  |
| Mean values of haemoglobin at day 7 | Mean HB was **10.75** | MD was 1.19 lower (1.94 lower to 0.44 lower) | - | 493 (2 observational studies) | ⨁◯◯◯ VERY LOW ^a^ |
| Percentage change in haemoglobin concentration from baseline (measured at day 7 | Mean change in Hb was **-2.13** | MD was 9.1 lower (12.55 lower to 5.65 lower) | - | 778 (5 observational studies) | ⨁⨁◯◯ LOW |
| ≥5% decline in Hb at day 7 | 430 per 1,000 | **723 per 1,000** (594 to 878) | **RR 1.68** (1.38 to 2.04) | 492 (2 observational studies) | ⨁◯◯◯ VERY LOW ^a^ |
| ≥10% decline in Hb at day 7 | 256 per 1,000 | **620 per 1,000** (474 to 810) | **RR 2.42** (1.85 to 3.16) | 492 (2 observational studies) | ⨁◯◯◯ VERY LOW ^a^ |
| ≥20% decline in Hb at day 7 | 85 per 1,000 | **284 per 1,000** (169 to 478) | **RR 3.36** (2.00 to 5.65) | 492 (2 observational studies) | ⨁◯◯◯ VERY LOW ^a^ |
| Rate of moderate Individuals with moderate (≤ 5g/l) anaemia at day 7 | 0 per 1,000 | **0 per 1,000** (0 to 0) | **RR 27.91** (1.15 to 674.51) | 493 (2 observational studies) | ⨁◯◯◯ VERY LOW ^a^ |
| Rate of moderate Individuals with severe (≤ 8 g/l) anaemia at day 7 | 48 per 1,000 | **216 per 1,000** (110 to 424) | **RR 4.50** (2.29 to 8.84) | 493 (2 observational studies) | ⨁◯◯◯ VERY LOW ^a^ |

| ***The risk in the intervention group** (and its 95% confidence interval) is based on the assumed risk in the comparison group and the **relative effect** of the intervention (and its 95% CI).   **CI:** Confidence interval; **MD:** Mean difference; **RR:** Risk ratio |
| --- |
| **GRADE Working Group grades of evidence** **High quality:** We are very confident that the true effect lies close to that of the estimate of the effect **Moderate quality:** We are moderately confident in the effect estimate: The true effect is likely to be close to the estimate of the effect, but there is a possibility that it is substantially different **Low quality:** Our confidence in the effect estimate is limited: The true effect may be substantially different from the estimate of the effect **Very low quality:** We have very little confidence in the effect estimate: The true effect is likely to be substantially different from the estimate of effect |

a. Imprecision rated very serious as small number of studies, smaller than the optimal information size
