# Supplementary material for: Safety of primaquine given to people with G6PD deficiency: systematic review of prospective studies
Source: Malar J. 2017 Aug 22;16:346. doi: 10.1186/s12936-017-1989-3 (PMC5568268; doi:10.1186/s12936-017-1989-3)
Supplement: Supplementary file 8 — Additional file 8. GRADE summary of findings table, mid-range dose (0.4–0.5 mg/kg) PQ in G6PD deficient compared to G6PD replete people. [file 12936_2017_1989_MOESM8_ESM.docx]

## Additional file 8: GRADE Summary of findings table, mid-range dose (0.4 to 0.5 mg/kg) PQ in G6PD deficient compared to G6PD replete people

| Outcomes | **Anticipated absolute effects^*^** (95% CI) | | Relative effect (95% CI) | № of participants  (studies) | Quality of the evidence (GRADE) |
| --- | --- | --- | --- | --- | --- |
|  | **Risk with Individuals given primaquine without G6PD deficiency** | **Risk with Individuals given primaquine with G6PD deficiency** |  |  |  |
| Mean values of haemoglobin at day 7 | Mean Hb was **11.48** | MD was 3.92 lower (4.93 lower to 2.91 lower) | - | 71 (1 observational study) | ⨁◯◯◯ VERY LOW ^a^ |
| Percentage change in haemoglobin concentration from baseline (measured at day 7 | Mean change in Hb was **-1.28** | MD was 4.99 lower (9.96 lower to 0.02 lower) | - | 113 (1 observational study) | ⨁◯◯◯ VERY LOW ^a^ |

| ***The risk in the intervention group** (and its 95% confidence interval) is based on the assumed risk in the comparison group and the **relative effect** of the intervention (and its 95% CI).   **CI:** Confidence interval; **MD:** Mean difference |
| --- |
| **GRADE Working Group grades of evidence** **High quality:** We are very confident that the true effect lies close to that of the estimate of the effect **Moderate quality:** We are moderately confident in the effect estimate: The true effect is likely to be close to the estimate of the effect, but there is a possibility that it is substantially different **Low quality:** Our confidence in the effect estimate is limited: The true effect may be substantially different from the estimate of the effect **Very low quality:** We have very little confidence in the effect estimate: The true effect is likely to be substantially different from the estimate of effect |

a. Imprecision rated very serious as small number of studies, smaller than the optimal information size
