# Supplementary material for: Safety of primaquine given to people with G6PD deficiency: systematic review of prospective studies
Source: Malar J. 2017 Aug 22;16:346. doi: 10.1186/s12936-017-1989-3 (PMC5568268; doi:10.1186/s12936-017-1989-3)
Supplement: Supplementary file 9 — Additional file 9. GRADE summary of findings table, low dose (0.1–0.25 mg/kg) PQ compared to placebo in G6PD deficient people. [file 12936_2017_1989_MOESM9_ESM.docx]

## Additional file 9: GRADE Summary of findings table, low dose (0.1 to 0.25 mg/kg) PQ compared to placebo in G6PD deficient people

| **Outcomes** | **Anticipated absolute effects^*^ (95% CI)** | | **Relative effect (95% CI)** | **№ of participants  (studies)** | **Quality of the evidence (GRADE)** |
| --- | --- | --- | --- | --- | --- |
|  | **Risk with Individuals with G6PD deficiency given placebo** | **Risk with Individuals with G6PD deficiency given Primaquine** |  |  |  |
| Percentage change in haemoglobin concentration from baseline (measured at day 7 | Mean change in Hb was **-6.28** | MD was 1.72 higher (1.89 lower to 5.34 higher) | - | 89 (2 RCTs) | ⨁⨁◯◯ LOW ^a^ |

| ***The risk in the intervention group** (and its 95% confidence interval) is based on the assumed risk in the comparison group and the **relative effect** of the intervention (and its 95% CI).   **CI:** Confidence interval; **MD:** Mean difference |
| --- |
| **GRADE Working Group grades of evidence** **High quality:** We are very confident that the true effect lies close to that of the estimate of the effect **Moderate quality:** We are moderately confident in the effect estimate: The true effect is likely to be close to the estimate of the effect, but there is a possibility that it is substantially different **Low quality:** Our confidence in the effect estimate is limited: The true effect may be substantially different from the estimate of the effect **Very low quality:** We have very little confidence in the effect estimate: The true effect is likely to be substantially different from the estimate of effect |

a. Imprecision rated very serious as small number of studies, smaller than the optimal information size

b. Inconsistency rated very serious as there was considerable heterogeneity in treatment effect estimates (I² > 75%)

c. Imprecision rated serious as small number of studies, smaller than the optimal information size
